# 3-D computer modelling of malunited posterior malleolar fractures: effect of fragment size and offset on ankle stability, contact pressure and pattern

**DOI:** 10.1186/s13047-017-0194-5

**Published:** 2017-03-11

**Authors:** Teresa Alonso-Rasgado, David Jimenez-Cruz, Michael Karski

**Affiliations:** 1 Bioengineering Research Group, School of Materials, The University of Manchester, Oxford Road, Manchester, M13 9PL UK; 2 Wrightington Hospital, Wigan and Leigh NHS Foundation Trust, Lancashire, UK

**Keywords:** Malunion, Tibiotalar joint, Posterior malleolar fracture, Finite element

## Abstract

**Background:**

The positioning of the fracture fragment of a posterior malleolus fracture is critical to healing and a successful outcome as malunion of a posterior malleolar fracture, a condition seen in clinical practice, can affect the dynamics of the ankle joint, cause posterolateral rotational subluxation of the talus and ultimately lead to destruction of the joint. Current consensus is to employ anatomic reduction with internal fixation when the fragment size is larger than 25 to 33% of the tibial plafond.

**Methods:**

A 3-dimensional finite element (FE) model of ankle was developed in order to investigate the effect of fragment size (6–15 mm) and offset (1–4 mm) of a malunited posterior malleolus on tibiotalar joint contact area, pressure, motion of joint and ligament forces. Three positions of the joint were simulated; neutral position, 20° dorsiflexion and 30° plantarflexion.

**Results:**

Compared to the intact joint our model predicted that contact area was greater in all malunion scenarios considered. In general, the joint contact area was affected more by section length than section offset. In addition fibula contact area played a role in all the malunion cases.

**Conclusions:**

We found no evidence to support the current consensus of fixing posterior malleolus fractures of greater than 25% of the tibial plafond. Our model predicted joint instability only with the highest level of fracture in a loaded limb at an extreme position of dorsiflexion. No increase of peak contact pressure as a result of malunion was predicted but contact pattern was modified. The results of our study support the view that in cases of posterior malleolar fracture, posttraumatic osteoarthritis occurs as a result of load on areas of cartilage not used to loading rather than an increase in contact pressure. Ankle repositioning resulted in increased force in two ankle ligaments. Our finding could explain commonly reported clinical observations.

## Background

The ankle joint is a synovial hinge joint permitting movement, plantarflexion and dorsiflexion of the foot, in a single plane [1]. It is composed of the distal articular surfaces of the tibia, the fibula and the talus guided by tendons and ligaments [2, 3]. The ankle joint is very stable due in the main to the congruency between the bony articulations of the joint supported by the ligaments of which the deltoid ligaments and the lateral collateral ligament and the syndesmotic ligament complexes play an important role in ankle dynamics [1]. The close fitting of the articular surfaces of the talar dome in the mortice formed by the tibial plafond, medial malleolus and lateral malleolus together with the supporting ligaments ensures the stability of the ankle complex [4, 5]. However, because the articulating surfaces of the talus are wider anteriorly compared to posteriorly, stability is greater during dorsiflexion than during plantarflexion. In a normal healthy joint at a neutral weight-bearing standing position, the calcaneus typically remains stationary while the talus moves freely on it [4].

The ankle joint is a major joint prone to injuries and conditions including fracture and arthritis. Recent studies have determined that the incidence of ankle fractures accounts for about 9% of all bone fractures [6, 7]. Although isolated fractures of the posterior malleolus, anatomically the bony protrusion that helps keep the talus in its position [8], are relatively rare [9], it has been determined that between 7 and 44% of all ankle fractures involve a posterior malleolus fracture component [9–11]. These fractures are classified as 44A3 or 44B3 under the Arbeitsgemeinschaft für Osteosynthesefragen, AO classification [12, 13]. It has been reported that larger fractures of the posterior malleolus can result in posterior subluxation of the talus and articular incongruity of the tibial plafond which, if not corrected can lead to posterior instability of the ankle and secondary traumatic arthritis [8, 14, 15] and whilst smaller fractures may not result in posterior subluxation, it has been suggested that they can still potentially lead to tibiotalar instability [16] and degenerative changes. Posttraumatic osteoarthritis resulting from fractures of the posterior malleolus can occur due to malreduction of the fracture fragment, cartilage damage due to the trauma and ankle instability [17].

Historically, nonsurgical treatment has generally been employed for stable, isolated posterior malleolus fractures and the decision to undertake surgical intervention has been taken based on fragment size [9, 16] commonly measured as the percentage of involvement of the distal tibial articular surface [9]. However, there is a lack of consensus amongst the clinical studies reported in the literature as to the fragment sizes which require internal fixation and this has been attributed to the difficulty in determining fragment size accurately using lateral radiographs, which have traditionally been used, and the need for standardization in measuring the functional outcomes of interventions [18]. A number of studies have recommended anatomic reduction with internal fixation when the fragment size is larger than 25 to 33% of the tibial plafond [9, 11, 18–25] although others have argued that the level of evidence as to whether internal fixation of posterior malleolar fractures leads to improved outcomes is not sufficiently high to support this recommendation [17, 19].

A number of experimental, biomechanical studies have been undertaken which have investigated the effect of posterior malleolar fracture on tibiotalar contact area, articular load distribution and contact stress [19, 23, 26]. Load characteristics of the ankle are complex [20]. When loaded, the ankle has a smaller contact surface area than both the knee and the hip [20] and it has been demonstrated that during normal walking the ankle joint is subjected to greater compressive forces than both these joints, with values up to and exceeding five times the body weight reported [11].

The review undertaken by van den Bekerom et al. [18] analysed and reported on several biomechanical studies investigating ankle instability amongst which were two cadaveric based studies that examined the change in contact area following posterior malleolar fracture [18, 23, 24]. In the biomechanical cadaver-based investigations performed by both Hartford et al. [23] and Macko et al. [24] tibiotalar contact area was found to decrease with increasing posterior malleolar fracture fragment size. In addition, load distribution patterns were found to alter, as fragment size increased confluence and load concentration also increased. Although stresses were not reported in these studies, it was suggested that the reduction in articular surface area could cause peak stresses and rates of post traumatic arthritis to increase [9]. In the unconstrained, dynamic cadaver model utilised by Fitzpatrick et al. [27] joint contact area was found to reduce slightly but significantly only following a 50% posterior malleolus osteotomy compared to the intact ankle. Although no increase in peak contact stress was observed following fracture, the contact stress pattern shifted anteromedially, particularly in dorsiflexion. The authors suggest that the shift causes stress on cartilage that usually sees little load and that this may contribute to posttraumatic arthrosis. Similarly, Vrahas et al [28] also reported seeing no elevation in peak tibiotalar peak stress in their cadaver based posterior malleolar fracture model but again a change in the articular stress distribution was found.

In the current study an investigation was undertaken into the effect of fragment size and offset of a malunited posterior malleolus on tibiotalar joint contact area, pressure and stability. A 3-dimensional finite element (FE) model of ankle was developed enabling virtual osteotomies to be undertaken in order to simulate fragment sizes ranging from 6 to 15 mm at offsets of between 1 to 4 mm. Three static positions of the ankle were considered: the neutral position, at 20° dorsiflexion and at 30° plantarflexion. The loads corresponding to those experienced during a two legged stand of a 70 kg subject were applied and tibiotalar contact area and pressure calculated for each fragment size, offset and ankle position combination.

The purpose of the study was threefold:

i. To investigate if there is a pathomechanical foundation for internal fixation of posterior malleolus fractures of greater than 25% of the tibial plafond and in doing so to help inform current consensus;
ii. To clarify the effect of posterior malleolar fracture fragment size and offset on ankle joint contact pressure and area, as contradictory results exist from previous cadaveric studies which has lead to alternative theories for the increased rates of post traumatic arthritis associated with fractures of the posterior malleolus.
iii. To investigate the effect of posterior malleolar fracture fragment length and offset on tibiotalar joint repositioning and the relationship with the forces exerted on the ankle joint ligaments.

Finite element analysis was chosen for the current study for a number of reasons. The Finite Element Method has become widely accepted as a valuable numerical tool for studying the biomechanics and the influence of mechanical forces on biological systems [29]. It is ideal for undertaking stress and strain analysis of bone and joints and load bearing implants [30]. It has been successfully used for the biomechanical analysis of major joints of human body including the shoulder [31], hip [32], spine [33] and knee [34]. Finite element analysis has a number of advantages compared with experimental and cadaveric studies, such as, amongst others, analyses are repeatable, there are no ethical considerations and the study parameters can be modified quickly and easily at low cost [29, 30]. They can be employed to help interpret clinical and experimental results and in cases when experiments are difficult [30, 35]. With cadaveric studies, obtaining significant numbers of specimens may be challenging from both availability and financial standpoints. In addition, cadaveric specimens are more likely to have undergone degenerative bone and ligament changes which can influence results [18]. A further advantage with a finite element based model is that due to its deterministic nature, complex statistical analyses of model generated data (predictions) is not required.

We found a lack of consensus among previous cadaveric ankle studies of ankle instability [23, 24, 27, 28]. A finding which had also has been reported with ankle and foot ankle impact injury studies [35]. Utilising our finite element model we investigated the effect of fragment size and offset of a malunited posterior malleolus on tibiotalar joint contact area, pressure and stability in an attempt to help clarify the cadaveric data available to-date. Model validation against experimental data is essential to ensure confidence in simulation predictions [36]. We validated our ankle model by comparing predicted contact area values and peak contact pressure magnitudes for the intact ankle with the results from a number of cadaveric studies. Once validated, a finite element model such as ours can be utilised to investigate scenarios where cadaveric or in-vivo experimentation and measurement is difficult, expensive or where a consensus has failed to be established from previous studies. In our case, we utilised our validated finite element ankle model to investigate a range of fracture scenarios as findings from previous cadaveric studies of ankle instability had failed to establish a consensus.

## Methods

This study investigates the effect on tibiotalar joint stability of malunion following a posterior malleolus fracture, in particular, the influence of fracture fragment size and offset on joint contact area and stress is examined. First, a 3D model of a healthy ankle joint was created. Virtual osteotomy procedures were then performed to simulate malunited posterior malleolar fractures with different fragment sizes and offsets. Fracture fragment sizes ranging from 6 to 15 mm at increments of 3 mm were simulated in combination with offsets of between 1 to 4 mm using a 1 mm increment. The loading condition corresponding to that of a 70 kg subject in a two leg stand position was applied. The ankle was considered in the neutral position, at 20° dorsiflexion and at 30° of plantarflexion and tibiotalar joint contact area and stress calculated in each position for all fracture fragment size—offset combinations.

The 3D finite element model was created from CT scan data of the healthy, left foot, and included the distal tibia, fibula, talus, calcaneus, cuboid and navicular bones, and the sixteen ligaments associated with the ankle joint. The procedure employed to obtain the 3-dimensional bone geometries of the ankle complex for use in the current analysis was based on that used previously by Alonso-Rasgado et al. [37] to generate bone geometries from CT scan data for inclusion in a 3-D numerical model of a hip joint.

**Fig. 1:**
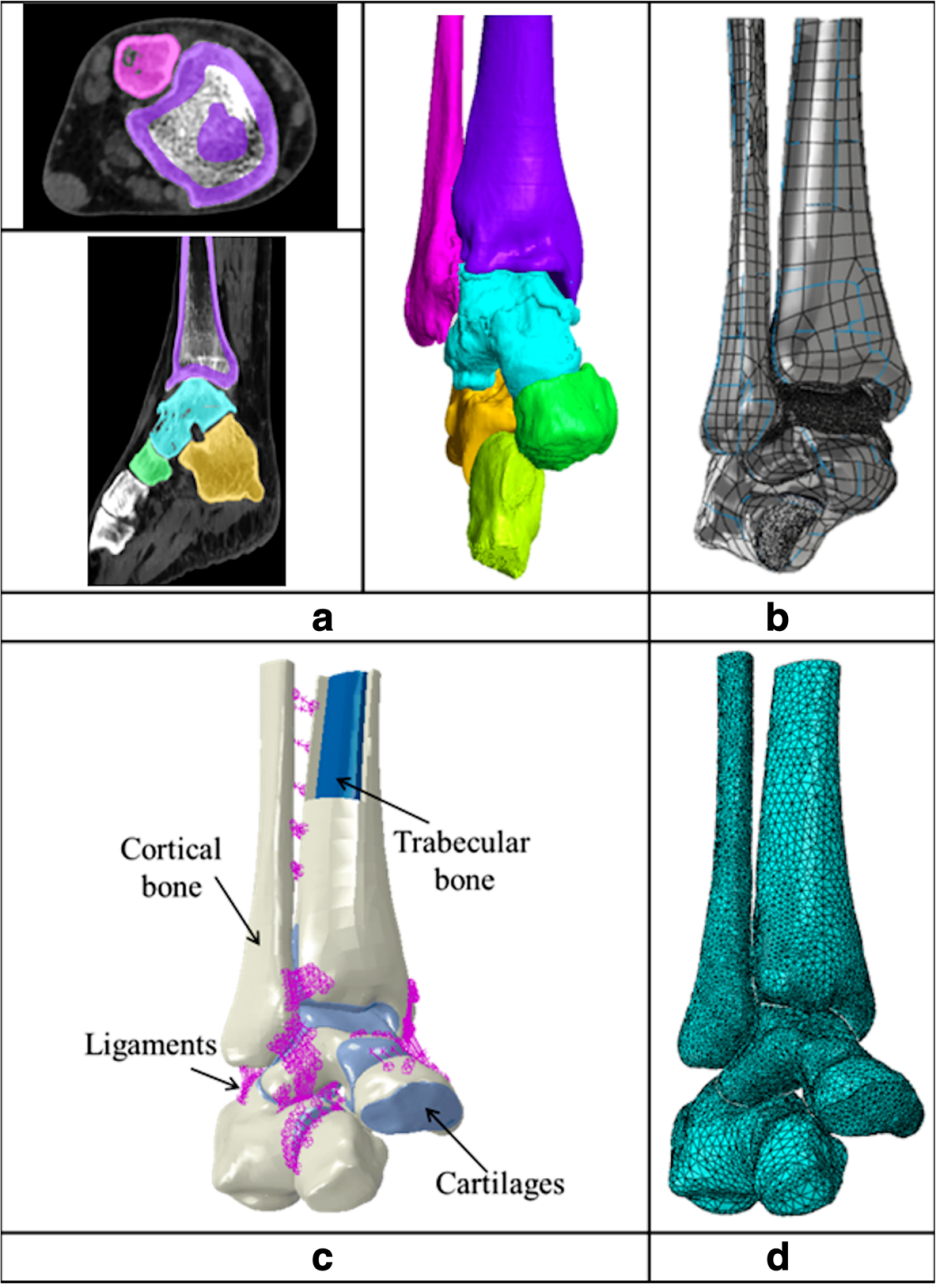
Model development process: **a** Colour mask in CT scan (ScanIP®); **b** 3D surface (SolidWorks®); **c** Ankle joint assembly materials (Abaqus CAE 6.10-1®); **d** Finite element representation (Abaqus CAE 6.10-1®)

First, DICOM data from the CT scans of the foot (Fig. 1a) were imported into an image processing software application enabling 3D surfaces of each of the bones included in the model to be produced through a segmentation process (Fig. 1b) [37]. The surface data were then exported from the image processing software and imported into SolidWorks® (Dassault Systèmes, SolidWorks Corp, Waltham, MA, USA), where solid models were generated from the surface data and the cartilage geometry and the insertion points for the ligaments (Fig. 1c) were added. The components were then imported and assembled in the Abaqus 6.13-3® FE analysis software (Abaqus, Inc., Dassault Systemes Simulia Corp, Providence, RI) where solid geometry pre-processing tools were employed to undertake the virtual osteotomies. Mesh generation and model analysis was then performed using the Abaqus software (Fig. 1d).

In order to perform the virtual osteotomies, a typical posterior malleolus fracture location was identified from the literature and confirmed by a surgeon. Virtual osteotomies were performed on the healthy ankle model (intact) creating additional models representing sixteen malunited posterior malleolus fracture scenarios. Four different section lengths (fracture fragment sizes) were simulated, 6, 9, 12 and 15 mm representing 18, 26, 35 and 44% of the total distal tibial articular surface, respectively. Each section length was modelled in combination with section offsets of 1, 2, 3 and 4 mm.

### Geometry

**Fig. 2:**
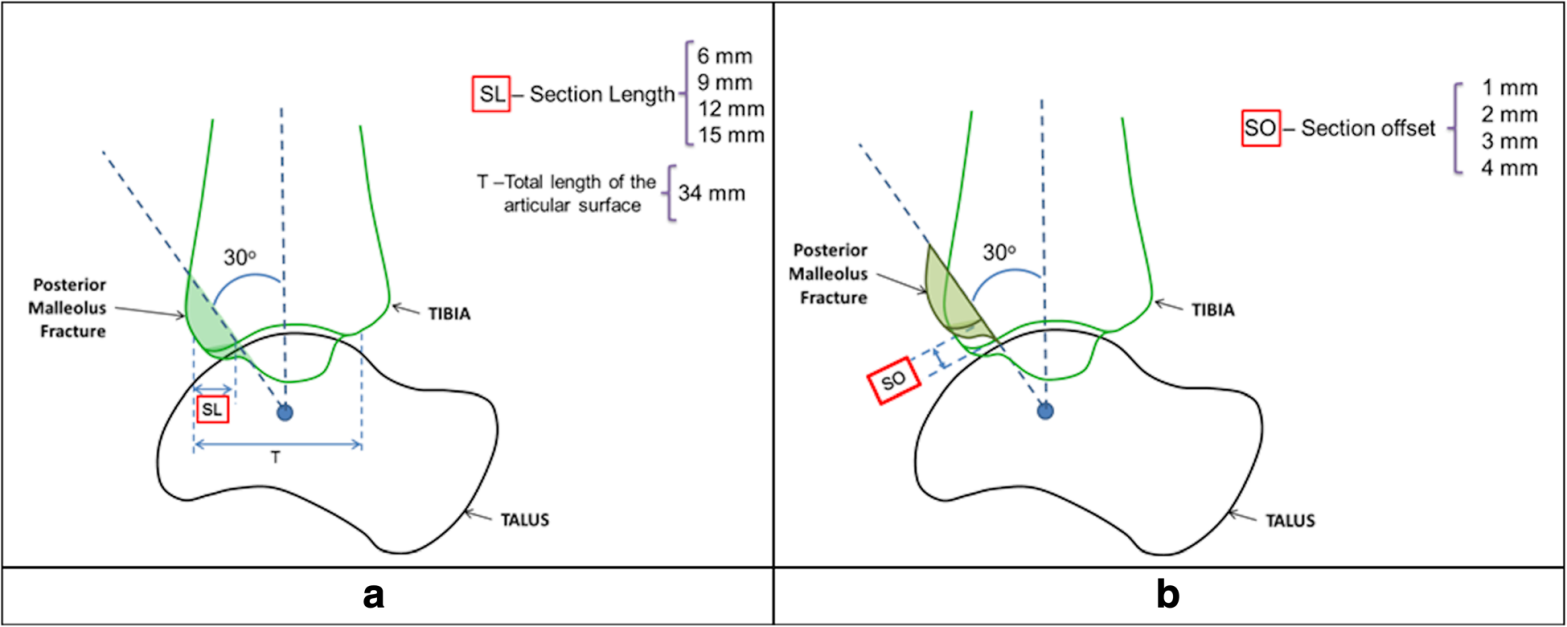
Parameters to perform the malunion virtual osteotomy: **a** Section length (SL) (**b**) Section offset (SO)

The geometries for the virtual malunion scenarios were created by partitioning the distal posterior malleolus section at an angle of 30° from a vertical line passing through the tibial shaft to the centre of rotation of the ankle joint. The partition generated was then translated horizontally to create the four required section lengths (SL) (Fig. 2a). The fracture fragment was repositioned upwards at an angle of 30° to the vertical to create the required section offsets (SO) (Fig. 2b). A constant thickness was assumed for the cartilage layers [38–40] and both the bone and cartilage geometries were meshed with type ‘C3D4’ solid linear 4-noded tetrahedral elements.

### Materials

**Fig. 3:**
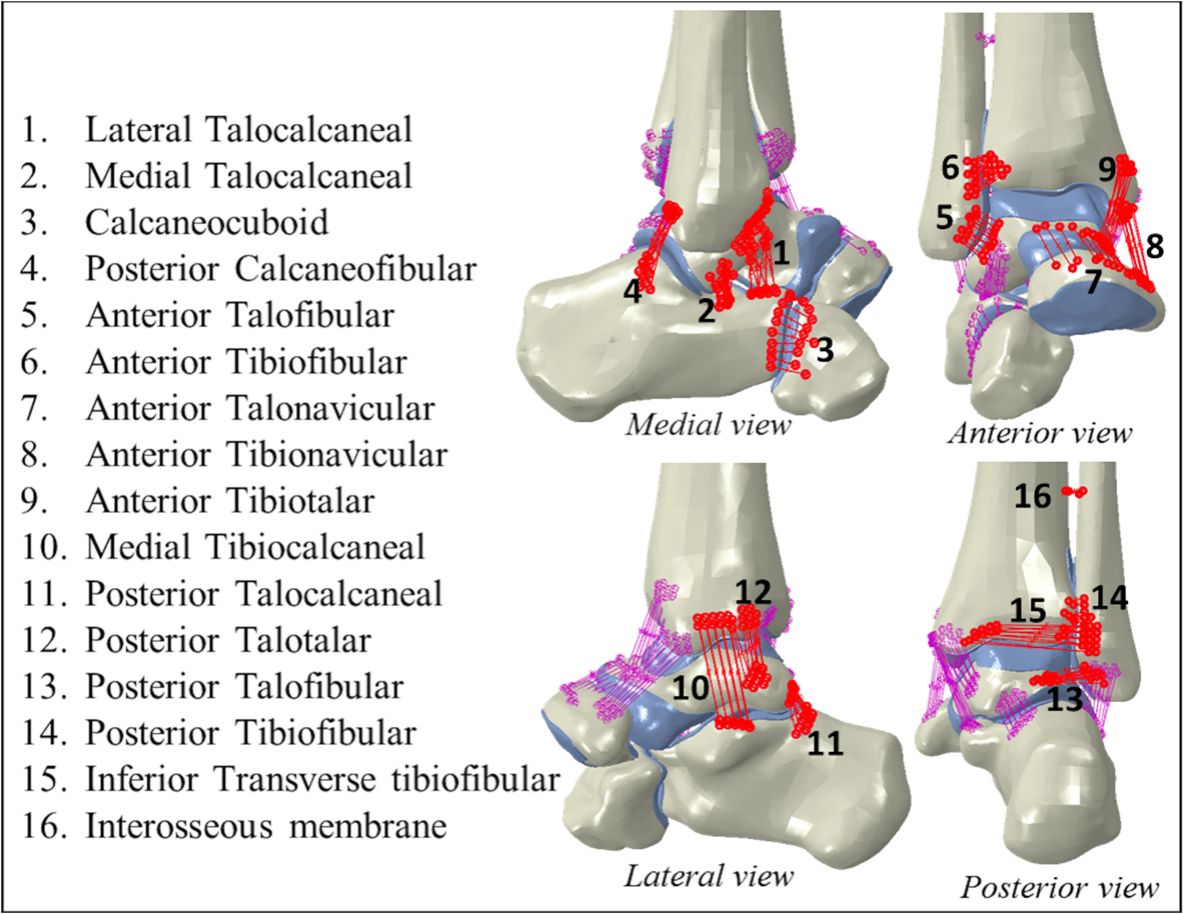
Ligaments considered in the model

The material properties employed in the models for the cortical and trabecular bone, cartilage and ligaments were derived from the literature. Bone and cartilage were assumed to behave as isotropic, elastic-plastic materials with properties as shown in Table 1 [37, 38, 41]. The ligaments of the ankle joint were represented using a number of spring elements in the Abaqus 6.13-3® software, inserted in the model at the relevant anatomical sites. Each ligament was modelled using 10 nonlinear spring elements of stiffness 15 N/mm [42–44] that only worked in tension (Fig. 3). Complete bonding was assumed between cartilage and cortical bone and between cortical and trabecular bone. The coefficient of friction of cartilage is low (μ < 0.0025) [45], therefore frictionless contact was assumed between cartilage surfaces in the model.

**Table 1:** Material properties

	Cortical bone	Trabecular bone	Cartilage
Density, ρ [t/mm^3^]	1.98e-9	4.3e-10	1.3e-9
Young’s modulus, E[MP]	17,000	477	12
Poisson’s ratio	0.3	0.3	0.4

### Boundary conditions

**Fig. 4:**
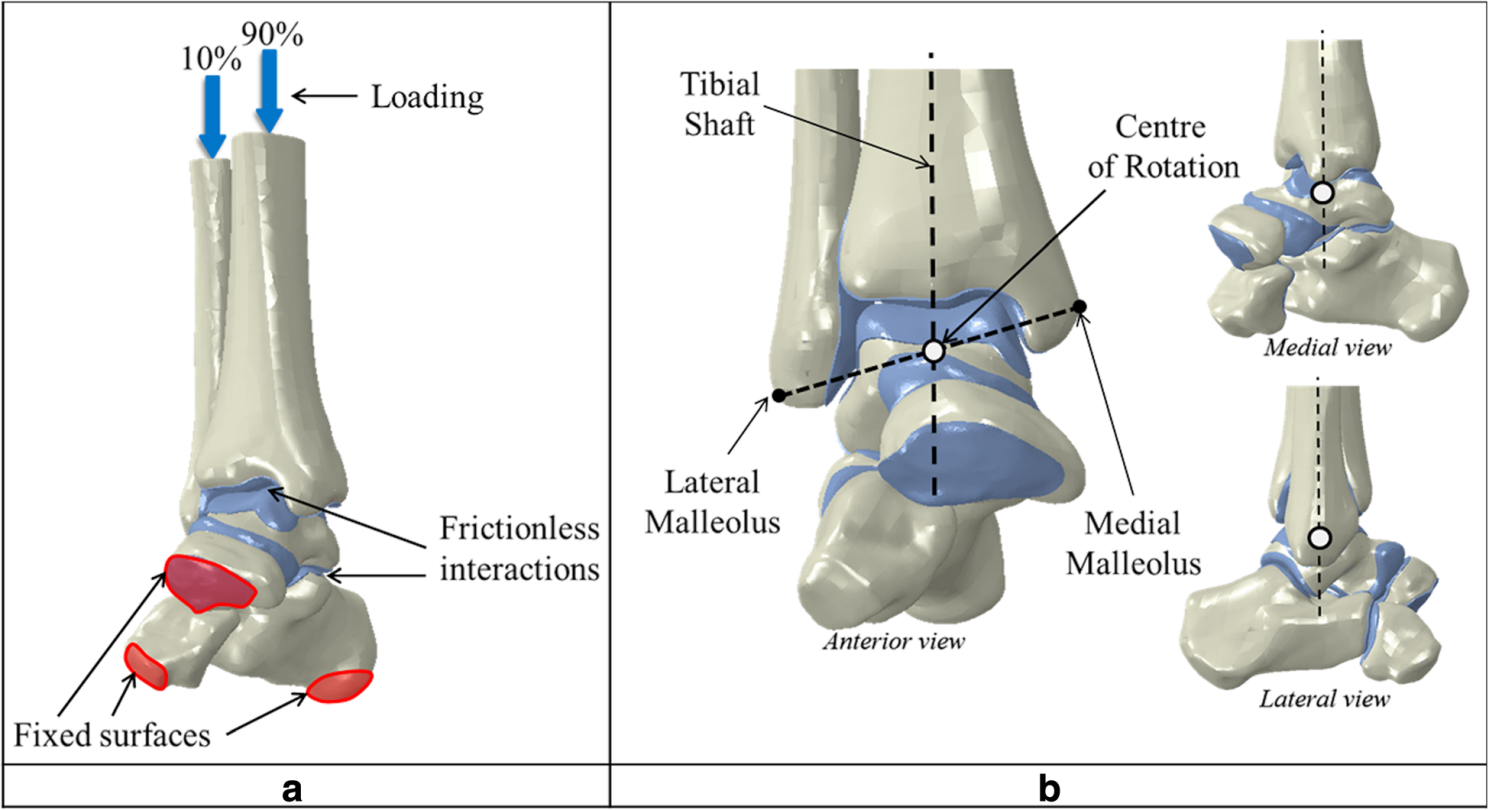
Boundary conditions. **a** Loads and surface interactions; **b** Centre of rotation

The ankle joint was simulated positioned in the neutral position, at 20° dorsiflexion and at 30° plantarflexion in the models and subject to loading corresponding to that resulting from a subject of 70 kg standing statically on two legs. Half the body weight (350 N) was applied to the proximal surfaces of the tibia and fibula, with the load split 90%:10% between the tibia and the fibula [46–48]. The lower surfaces of the calcaneus, cuboid and navicular bones were fully fixed in terms of displacement and rotation (Fig. 4a).

The centre of rotation of the ankle joint was located inside the talus by determining the intersection of an imaginary line running from the lateral to the medial malleolus and a vertical line running through the middle of the tibial shaft. The centre of rotation was fixed in all translational axes but rotation around the medial-lateral axis (Fig. 4b) was permitted.

The tibia and fibula were connected by a kinematic coupling which ensured that they worked together and coupled motion was achieved. Motion of the tibia and talus were restricted using 3-D connector elements in order to ensure realistic simulation and motion of the joint [38, 49]. These types of elements are useful for connecting and simulating the interaction between two different components of a model. A Cylindrical connector was used to link the tibia and the talus. This connector provided a damping type mechanism, allowing rotation and displacement around a local longitudinal axis. A Cardan connector was used to link the talus and tibia enabling dorsiflexion and plantarflexion motions to be simulated as well as inversion and eversion.

Motion was applied at the centre of rotation of the ankle; with the ankle starting in the neutral standing position, the tibia and talus were rotated anteriorly and posteriorly around the medial-lateral or talocrural axis to simulate the dorsiflexion and plantarflexion motions, respectively.

### Model validation

The 3-dimensional finite element (FE) model was corroborated by comparing predicted contact area values and peak contact pressure magnitudes for the intact ankle with the results from a number of cadaveric studies [22, 23, 27, 50].

Our intact ankle model predicted a total tibial plafond contact area of 240 mm^2^ under a loading condition corresponding to that of a 70 kg subject undertaking a two leg stand for ankle positions from 20^o^ dorsiflexion to 30^o^ plantarflexion. This value is comparable to values determined from cadaveric studies, including those of Brown et al. [22] (196.4 ± 64.4 mm^2^), Kimizuka et al. [50] (229 mm^2^), Hartford et al. [23] (256–426 mm^2^) and Fitzpatrick et al. [26] (300–400 mm^2^) for intact ankle when considering proportionate loading.

In addition, the peak contact pressure predicted by our intact ankle model was 6 MPa; this compares well with the values reported by Kimizuka et al. [50], who determined a mean peak contact pressure value of 4.4 MPa from their study, Vrahas et al. [28] who reported a peak contact stress of 6.7 MPa and Fitzpatrick et al. [27] who found peak contact stress values of 7–9 MPa.

## Results

This study has described a 3-D finite element model of the ankle that can predict the contact area and contact pressure in the tibiotalar joint region for different ankle positions. Using this model, we have investigated the contact area and contact pressure of the joint when malunion occurs in a posterior malleolar fracture for a finite combination of section length and section offsets for the ankle in the neutral position, 20° dorsiflexion and 30° plantarflexion. The contact area defined is the articulation area of the distal tibial plafond against the surface of the talus.

### Contact area calculation

For the purpose of analysing the outcomes from this study, the contact area on the tibiotalar joint is reported as it is the affected area by the malunion in a posterior malleolar fracture. The contact area was calculated from the coordinates of the nodes that were identified as being on the tibiotalar cartilage surfaces that are in contact in the neutral position and during dorsiflexion and plantarflexion for the intact and malunion models.

### Contact area

**Fig. 5:**
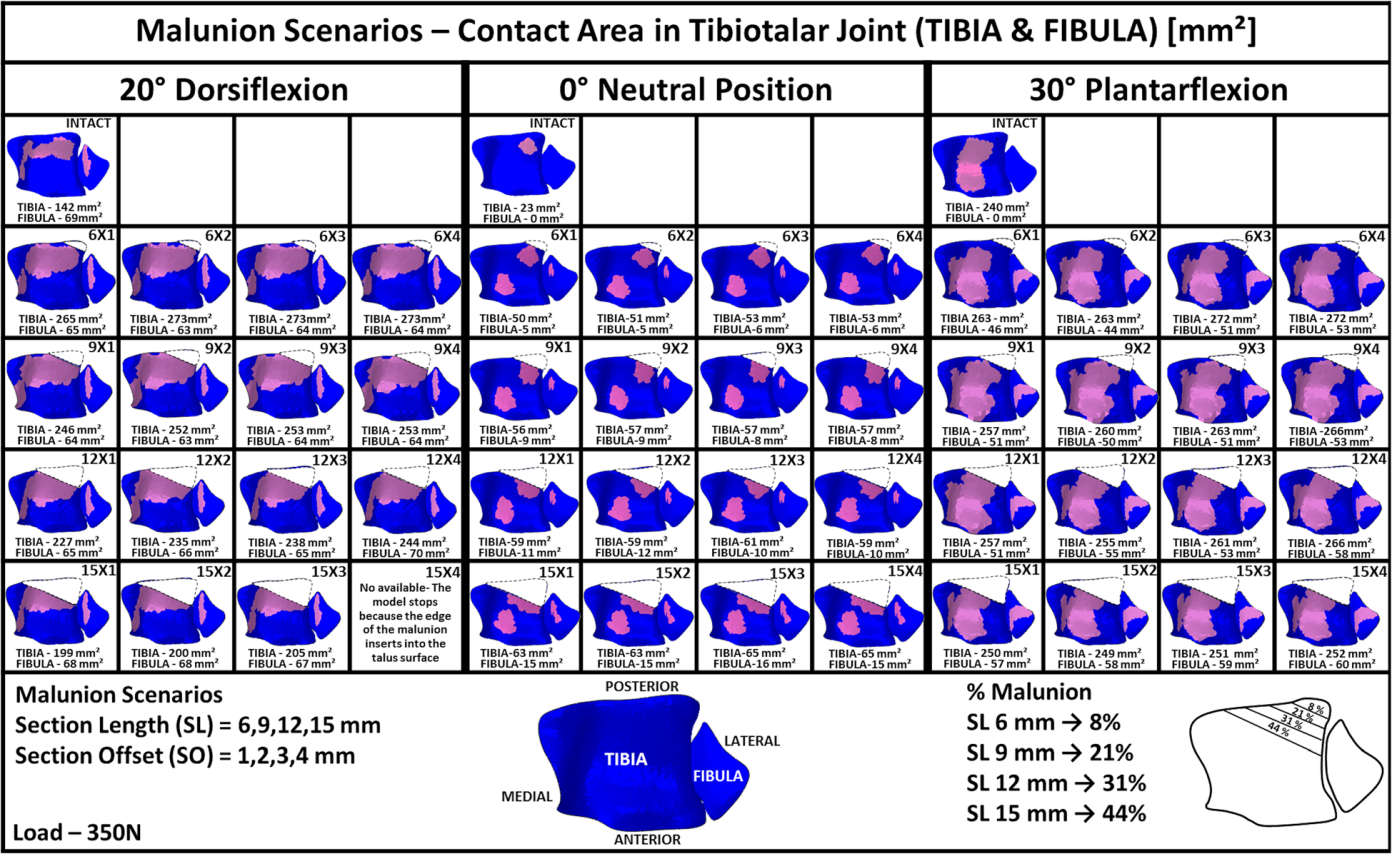
Contact areas on the Tibia and Fibula for intact and malunited posterior malleolar fracture scenario

**Fig. 6:**
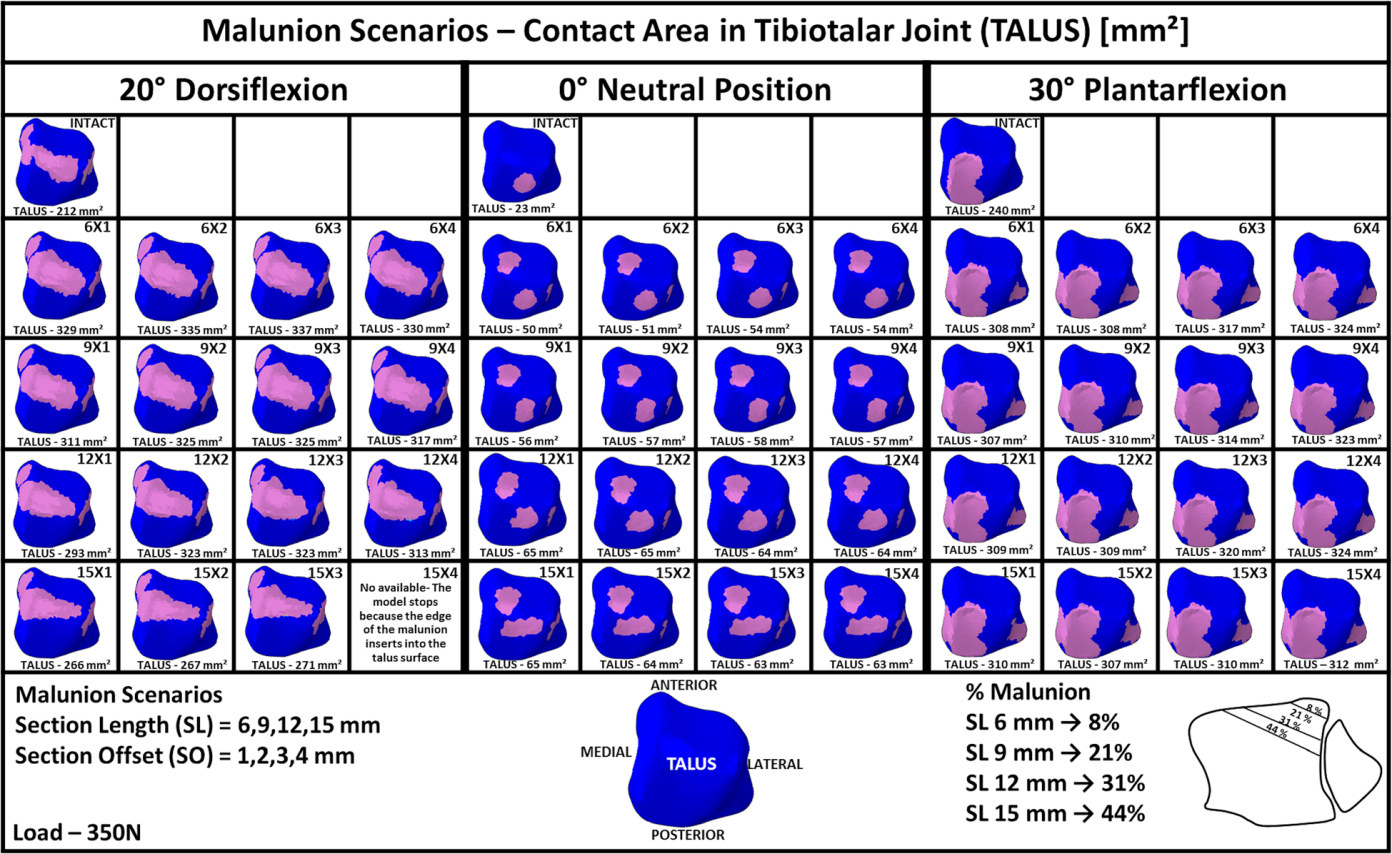
Contact areas on the Talus for intact and malunited posterior malleolar fracture scenarios

Figure 5 shows the predicted contact areas on the tibia and fibula in the tibiotalar joint for the intact and malunited posterior malleolar fracture scenarios for the three ankle joint positions considered: neutral position, 20^o^ dorsiflexion nd 30^o^ of plantarflexion. Figure 6 shows the corresponding contact areas on the talus for the same conditions.

#### Intact

It can be seen upon inspection of Fig. 5 that in the intact model, with the joint positioned in the neutral position, the contact area of the tibia was concentrated in the posterior-lateral region of the tibia; no contact was predicted on the fibula, indicating it does not play a role in this case.

For the intact ankle joint, tibia contact area increased in both dorsiflexion and plantarflexion compared to the neutral position. The maximum joint contact area determined, 240 mm^2^, occurred with the ankle positioned at 30^o^ of plantarflexion. In plantarflexion, contact area increased anterior-medially compared to neutral position. For the neutral position, no contact was predicted on the fibula. In dorsiflexion, contact area was concentrated posterior-medially on the tibia. In addition, on the fibula, a contact area was also predicted for this position (69 mm^2^).

The contact areas on the talus for the intact ankle for these scenarios, essentially the mirror image of those determined for the tibia and fibula, are shown in Fig. 6.

### Fracture

#### Neutral position

In the fracture scenarios with the ankle in the neutral position, tibia contact area increased with section length with additional areas of contact being established anterior-medially on the tibia and also on the fibula. In contrast, contact pattern and area values were relatively invariant with section offset. The contact area in the posterior-lateral region of the tibia included the fracture site.

#### Dorsiflexion

In the fracture scenarios with the ankle in 20^o^ dorsiflexion, tibia contact area was greater than for the intact ankle. Compared to the intact ankle, contact area grew anterior and medially in the fracture scenarios. The greater contact area occurred for smaller section length (6 mm); following this tibia contact area reduced as section length increased. This was as a result of the tibia malunion moving over the talus dome in dorsiflexion; with increasing fracture fragment, less of the intact tibia is available for contact; this is shown in Fig. 5 where it can be clearly seen that contact area reduces posteriorly as fracture fragment length increases. Section offsets of 2 mm and greater lead to greater tibia contact areas for all section lengths considered. Fibula contact area remained relatively invariant in the fracture scenarios compared to the intact ankle in dorsiflexion so overall, total joint contact area reduced as fragment size increased beyond 6 mm, as can be seen from the corresponding talus contact area predictions shown in Fig. 6. Note, the predictions for section length 15 mm, section offset 4 mm at 20^o^ dorsiflexion are unavailable as the simulation stopped due to the edge of the malunion inserting into the talus surface, signifying joint instability for this case.

#### Plantarflexion

In the fracture scenarios with the ankle in 30^o^ plantarflexion, tibia contact area was greater than for the intact ankle. Compared to the intact ankle, contact area grew medially in the fracture scenarios; in addition, contact was predicted on the fibula, unlike the intact ankle joint case. As was the case for dorsiflexion, the greater tibia contact area occurred for the smaller section length (6 mm) for all section offsets considered; following this tibia contact area reduced as section length increased. However, unlike the dorsiflexion case, total joint contact area remained relatively constant (307–324 mm^2^) because although tibia contact area reduced with increasing section length, fibula contact area grew; this is confirmed by the talus contact area predictions shown in Fig. 6. Section offsets of greater than 2 mm tended to lead to an increase in joint contact area for all section lengths considered.

### Contact pressure

Predicted peak joint contact pressure in the intact model was greatest in plantarflexion (6 MPa) and lowest in dorsiflexion (2.4 MPa). For the malunion fracture scenarios, peak contact pressure was lower compared to the intact for the neutral and plantarflexion positions but higher in dorsiflexion. In dorsiflexion, peak contact pressure increased with section length whilst being relatively invariant to section offset. In the neutral position, peak contact pressure decreased with increasing section length but did not vary significantly with section offset. In plantarflexion, peak contact pressure did not vary significantly with either section length or offset. For the fracture scenarios, the greatest peak contact pressure, 6 MPa, occurred for the largest section length 15 mm, with the ankle positioned in dorsiflexion. Compared to the intact ankle, overall, maximum peak contact pressure did not rise for the malunion fracture cases.

### Ankle repositioning and effect on ligament force

**Fig. 7:**
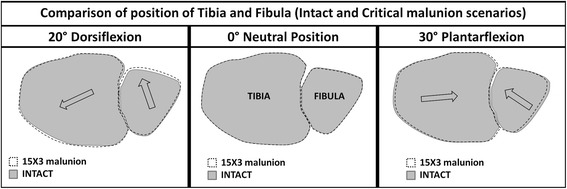
Reposition of the Tibia and Fibula after fracture malunion. Arrows show the direction of the reposition of the malunion scenario over the intact scenario in the transversal plane

We analysed the effect of malunion fracture length and offset on ankle repositioning including the forces on ankle joint ligaments. The effect on the 16 ligaments considered in the model, as shown in Fig. 3, was investigated. Ankle repositioning is shown in Fig. 7 where the repositioned ankle is shown, denoted by the dotted outline, relative to the original position, and for the most critical cases of malunion for which predictions were obtained for the three ankle positions. It can be seen upon inspection of this figure that in the neutral position, malunion caused no discernible movement or alteration in ankle position.

**Fig. 8:**
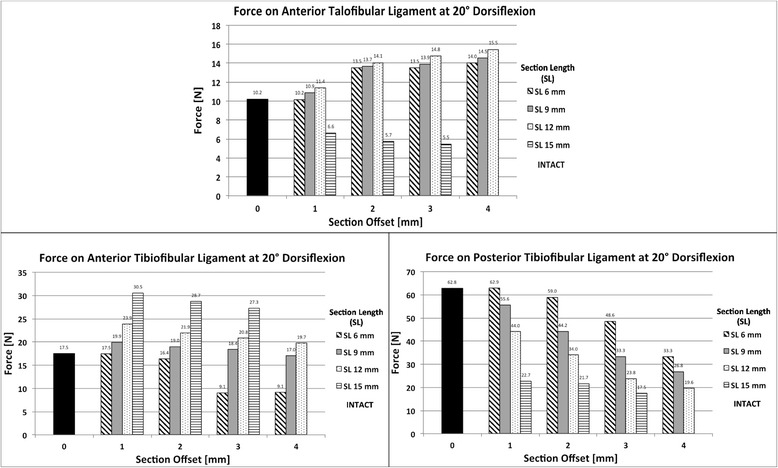
Force on the ligaments affected by the malunion. **a** Anterior Talofibular ligament. **b** Anterior Tibiofibular Ligament. **c** Posterior Tibiofibular ligament

In plantarflexion, malunion caused the joint to translate both anterior and medial. In dorsiflexion, the joint translated posterior and medial for a 15 mm fracture fragment and 3 mm section offset. Only in dorsiflexion did ankle joint translation following malunion have a notable effect on joint ligaments. Three ligaments, posterior tibiofibular, anterior talofibular and anterior tibiofibular, were affected. Whilst malunion generally caused forces to reduce in posterior tibiofibular, it resulted in force increases in both the anterior talofibular and anterior tibiofibular ligaments compared to the intact, as can be seen in Fig. 8. Forces in the anterior talofibular ligament were raised by up to 52% compared to the intact while those in anterior tibiofibular were increased by as much as 75%.

## Discussion

The effect of section length and section offset of a posterior malleolar fracture on joint contact area was investigated in this study. Our model predicted that, compared to the intact joint, contact area was greater in all malunion scenarios considered. Some earlier cadaveric studies (1991–1993) [23, 24] reported that tibiotalar contact area decreased with increasing fracture fragment. Macko et al. [24] reported reductions in tibiotalar contact area of between 4 and 35% for fracture fragments of 25, 33 and 50% in their cadaveric model whilst Hartford et al. [23] found contact area reduced by 4–22% for fracture fragments of 25–50%. However, in a more recent study (2004), Fitzpatrick et al. [27] reported no significant change of contact area following the creation of a 50% posterior malleolar fracture. In the earlier cadaver studies [23, 24], pressure sensitive film was employed to measure ankle joint contact pressure under static loading conditions, whereas the more recent study by Fitzpatrick et al.[27], with which our model predictions closely agree, employed a dynamic unconstrained model which provided for a more realistic loading pattern. In the cadaveric studies, ankle joint arthroplasty was required for insertion of the pressure sensitive film and this, combined with the presence of the film during the tests could potentially have altered ankle kinematics and joint stress, accounting for additional differences with cadaver study results and our model predictions [27]. In addition, soft tissue constraints were removed from some of the cadaveric ankles and the cadaveric specimens are more likely to have undergone degenerative bone and ligament changes than the patients presenting with posterior malleolar fractures in the clinical setting [18].

From our study we also found that, in the neutral position, joint contact area increased with fracture section length, and whilst total joint contact area remained relatively constant regardless of section length and offset in plantarflexion, in dorsiflexion, joint contact area reduced as fragment size increased beyond 6 mm (8% fracture). Overall, section length had a greater effect on joint contact area than section offset. The model predicted joint instability in only one of the scenarios considered, for a section length of 15 mm, section offset 4 mm in dorsiflexion.

Large fractures of the posterior malleolus have been implicated in posttraumatic osteoarthritis [8, 14, 15] whilst smaller fractures, it has been suggested, may lead to tibiotalar instability [16] and degenerative changes. It was initially thought that posttraumatic osteoarthritis occurred as a result of a reduction in joint contact area and a corresponding increase in contact pressure [18]. However, this has been questioned more recently following a number of biomechanical studies of posterior malleolar fractures [18, 27]. In addition, several studies have reported no elevation of peak contact pressure following fracture in biomechanical models [27, 28]. The results from our study concur with these two experimentally determined observations; our model predicted no decrease in tibiotalar joint contact area and no overall elevation of peak contact pressure as a result of malunion following posterior malleolar fractures of 6–15 mm. However, as has been reported in a number of cadaveric studies [27, 28], our model predicted a definite modification to contact pattern following fracture. In the neutral position, following fracture, additional areas of contact were established anteriorly and medially on the tibia and also on the fibula. In plantarflexion, tibia contact area grew medially and fibula contact area also increased. In dorsiflexion, tibia contact area grew anterior and medial in the fracture scenarios, a finding also reported by Fitzpartick et al. [27]. These results support the view that in cases of posterior malleolar fracture, posttraumatic osteoarthritis occurs as a result of a change in joint contact pattern which causes load on areas of cartilage not used to loading and not, as previously thought, due to an increase in contact pressure.

Current consensus suggests internal fixation for posterior malleolus fractures of greater than 25% of the tibial plafond [17]. We found no evidence to support this from our study. Our model predicted instability only with the highest level of fracture in a loaded limb at an extreme position of dorsiflexion. This concurs with a number of previous studies that determined that following fracture, posterior stability is likely to be maintained so long as lateral ligaments remain intact [27, 51, 52]. The lack of instability (except in one extreme case), combined with the fact no increase in peak ankle joint contact pressure was found for the fracture scenarios points to posterior malleolus fractures being relatively benign in nature. This supports the findings of an earlier review of the literature on the biomechanical and clinical evaluation of posterior malleolar fractures [18] and a recent study of the long-term outcome of 886 combined cases of posterior malleolar fractures from 1978 to 2014 undertaken by Veltman et al [17]. Veltman et al found that conservative treatment of posterior malleolar fractures showed comparable results on long-term outcome to surgical treatment, with no evidence to support the current consensus of fixing posterior malleolus fractures of greater than 25% of the tibial plafond.

When analysing ankle repositioning in the fracture scenarios considered, the model determined that force increased significantly in two ankle ligaments, anterior talofibular and anterior tibiofibular, in dorsiflexion. It is not known if the increased forces in these ligaments would result in clinical symptoms for patients.

Our finite element model is subject to some limitations and simplifications typically associated with complex numerical analyses in orthopaedic-related biomechanics. However, we validated our intact ankle model by comparing predicted contact area values and peak contact pressure magnitudes with the results from a number of cadaveric studies, which suggests that the simplifications and assumptions adopted in our model did not introduce significant error. Ligament and cartilage (soft tissue) behaviour was assumed to be linear elastic. Ligaments typically exhibit non-linear viscoelastic behaviour. To model this behaviour requires the specification of a significant number of parameters, for which accurate data are not readily available [53]. In addition, ligaments are known to operate close to the linear region [54], therefore a purely linear representation would enable good accuracy to be achieved. A neo-Hookean hyperelastic model is generally considered to provide a more accurate representation of cartilage behaviour. However, in finite element studies concerned primarily with joint contact stress and area analyses, little difference has been reported between predictions obtained assuming linear cartilage behaviour compared to neo-Hookean hyperelastic behaviour [55]. We analysed a single ankle specimen. The purpose of the study was to compare, for a given anatomy, ankle joint contact pressure and area for different malleolar fracture fragment sizes and offsets, not to quantify ankle joint pressure and contact area for a range of ankle specimens, therefore the use of a single ankle specimen was appropriate for this purpose.

For future research, we suggest applying our model to clinical case data. For example, by utilising our ankle model in conjunction with a randomized controlled trial it may be possible to establish a pathomechanical foundation for applying fixation for particular levels of fracture backed by clinical results.

## Conclusions

The model predicted joint instability only with the highest level of fracture in a loaded limb at an extreme position of dorsiflexion. No increase of peak contact pressure as a result of malunion was predicted but contact pattern was modified.

These results suggest that posterior malleolus fractures are relatively benign in nature. We found no evidence to support the current consensus of fixing posterior malleolus fractures of greater than 25% of the tibial plafond. In addition, the results of our study support the view that in cases of posterior malleolar fracture, posttraumatic osteoarthritis occurs as a result of a change in joint contact pattern which causes load on areas of cartilage not used to loading and not, as previously thought, due to an increase in contact pressure.

Ankle repositioning resulted in increased force in two ankle ligaments. Our finding could explain commonly reported clinical observations.
